# Genome-Wide Identification and Expression Profiling of the BZR Transcription Factor Gene Family in *Nicotiana benthamiana*

**DOI:** 10.3390/ijms221910379

**Published:** 2021-09-26

**Authors:** Xuwei Chen, Xinyang Wu, Shiyou Qiu, Hongying Zheng, Yuwen Lu, Jiejun Peng, Guanwei Wu, Jianping Chen, Shaofei Rao, Fei Yan

**Affiliations:** 1 State Key Laboratory for Managing Biotic and Chemical Threats to the Quality and Safety of Agro-Products, Key Laboratory of Biotechnology in Plant Protection of Ministry of Agriculture and Zhejiang Province, Institute of Plant Virology, Ningbo University, Ningbo 315211, China; 1811075003@nbu.edu.cn (X.C.); xy_wu@zju.edu.cn (X.W.); qiushiyoudyzx@126.com (S.Q.); zhenghongying@nbu.edu.cn (H.Z.); luyuwen@nbu.edu.cn (Y.L.); pengjiejun@nbu.edu.cn (J.P.); wuguanwei@nbu.edu.cn (G.W.); jianpingchen@nbu.edu.cn (J.C.); 2 College of Life Science, China Jiliang University, Hangzhou 310058, China

**Keywords:** Brassinazole-resistant (BZR) transcription factor, *Nicotiana benthamiana*, genome-wide expression analysis, abiotic stress

## Abstract

Brassinazole-resistant (BZR) family genes encode plant-specific transcription factors (TFs), play essential roles in the regulation of plant growth and development, and have multiple stress-resistance functions. *Nicotiana benthamiana* is a model plant widely used in basic research. However, members of the BZR family in *N. benthamiana* have not been identified, and little is known about their function in abiotic stress. In this study, a total of 14 BZR members were identified in the *N. benthamiana* genome, which could be divided into four groups according to a phylogenetic tree. *NbBZRs* have similar exon-intron structures and conserved motifs, and may be regulated by cis-acting elements such as STRE, TCA, and ARE, etc. Organ-specific expression analysis showed that *NbBZR* members have different and diverse expression patterns in different tissues, and most of the members are expressed in roots, stems, and leaves. The analysis of the expression patterns in response to different abiotic stresses showed that all the tested *NbBZR* members showed a significant down-regulation after drought treatment. Many *NbBZR* genes also responded in various ways to cold, heat and salt stress treatments. The results imply that NbBZRs have multiple functions related to stress resistance.

## 1. Introduction

The plant steroid hormones, Brassinosteroids (BRs), play an important role in regulating plant growth and development, and biotic and abiotic stress responses [1]. In the past 20 years, the signal transduction pathway of BR has been well resolved [2,3,4,5]. The BR receptor Brassinosteroid insensitive 1 (BRI1) is a leucine-rich, repeated, receptor-like kinase (LRR RLK) located in the plasma membrane. When BR molecules are detected, it recruits another LRR RLK BRI1- associated receptor kinase (BAK1) to form a receptor complex. The fully activated BRI1/BAK1 complex then triggers a series of downstream phosphorylation events, which ultimately lead to the regulation of a large number of genes [2,6,7,8]. Brassinazole resistant 1 (BZR1) and Brassinosteroid insensitive 1-ethyl methanesulfonate-suppressor 1 (BES1) are two downstream transcription factors (TFs) belonging to the Brassinazole-resistant (BZR) TF family. They can be dephosphorylated by protein phosphatase when BR is detected [9]. Dephosphorylated BZR1 and BES1 accumulate in the nucleus and directly bind to cis-elements to regulate plant growth and development [10].

The BZR gene family encodes a novel, plant-specific TF, which can directly mediate and regulate BR signaling [11]. In Arabidopsis, the BZR gene family includes *BZR1, BES1*, and *BES1/BZR1 homolog1 (BEH1)-BEH4*, which are highly similar to *BZR1/BES1* [8,10,12]. Both BZR1 and BES1 have a basic helix-loop-helix (bHLH) DNA binding motif at the N-terminus, which is a highly conserved domain in the entire family, but their functions are different [12]. BZR1 binds to a BR response element (CGTGT/CG motif) to inhibit the BR-biosynthetic gene expression [13], while BES1 binds to the E box (CANNTG sequence) to activate BR-induced gene expression [14]. In addition to the highly conserved N-terminal domain, the central region of the BZR protein usually has 22–24 predicted Glycogen synthase kinase-3 (GSK3) family phosphorylation sites. Some proteins also contain putative PEST motifs involved in protein degradation [12].

The tolerance of plants to adversity, such as water stress, temperature, or changes in soil salinity, depends on their ability to switch between growth activation and inhibition under adverse conditions [15,16]. The abscisic acid (ABA) signaling pathway is a key method to control environmental stress responses [17,18] but it appears that BRs mediate the adaptation to drought, cold, heat, and salinity either by crosstalk with the ABA pathway or independently of it. The regulation of the BZR transcription factors in Arabidopsis is an important point of convergence between BR and stress responses [1,19].

Several mechanisms that inhibit BES1 activity during stress are described. Under drought stress, BES1 inhibits BR-regulated growth through DSK2-mediated autophagy degradation [20]. In addition to regulating the abundance of the BES1 protein, drought also affects BES1 activity through its interaction with Responsive to desiccation 26 (RD26). RD26 is transcriptionally induced during drought, and then RD26 inhibits BES1 and promotes the drought response. At the mechanistic level, these two TFs may interact to form a heterodimer, which synergistically binds to G-Box (CACGTG) promoter elements. BES1 and RD26 have opposing activities, and RD26 inhibits BES1 activity by interacting with BES1 on a common promoter element [21,22]. TINY is an Apetala2/ethylene responsive factor TF. In drought stress, TINY is also induced to antagonize BES1, inhibit the growth regulated by BR and promote the expression of drought-responsive genes [23]. WRKY46, WRKY54 and WRKY70 can directly interact with BES1 to promote BR-regulated plant growth, while suppressing drought-induced, genome-wide transcripts to inhibit drought tolerance. However, the protein level of WRKY54 decreases during drought stress to induce drought tolerance in plants. Therefore, the degradation of BES1-interacting transcription factors during drought is another mechanism through which plants respond to drought [24].

Recent studies also show that BZR transcription factors are involved in regulating growth response when the temperature fluctuates. Low temperature treatment can also induce the dephosphorylation of BZR1 and BES1. The unphosphorylated forms of BZR1 and BES1 can promote the expression of C-repeat/dehydration-responsive element binding factor1 (CBF1) and CBF2, which positively regulates cold-stress responses. The study by Li et al. showed that, under non-domesticated and domesticated conditions, the overexpression of BIN2 could cause a hypersensitivity to freezing stress, while the triple mutants of *bin2–3 bil1 bil2*, and the gain-of-function mutants *bzr1–1D* and *bes1-D*, have an enhanced tolerance to freezing stress [25]. When the temperature increases, BZR1 accumulates in the nucleus and induces the expression of growth promoting genes, which can regulate thermalmorphogenesis directly or by binding to the promoter of *Phytochrome interacting factor 4* (*PIF4*) [26,27].

High salt can also cause root growth arrest by inhibiting the accumulation of BZR1 in the nucleus and the subsequent BR signaling function [28]. The exogenous application of BR can help plants cope better with high salt conditions by regulating the output of BR and ethylene signals [19].

BZR TF is also shown to play an important role in participating in multiple stress response pathways in other species. In wheat, the BES/BZR family transcription factor TaBZR2 plays an active role in drought response by activating *T. aestivum Glutathione S-transferase-1* (*TaGST1*) and mediates the dialogue between BR and drought signaling pathways [29]. Li et al. found that the *BZR* gene in legumes can significantly respond to drought and salt stress [30]. In *Brassica rapa*, members of the BrBZR TF family may be involved in the regulation of stress-related activities [31]. Manoli et al. found that maize BZR TFs not only play a role in regulating plant physiology and morphology, but also have stress signal activity [32]. Thus, these transcription factors play an active role in the BR signal transduction in many plants. However, members of the BZR TF family in the important experimental plant, *Nicotiana benthamiana*, have not been identified, and their related functions in stress resistance are unreported. In this study, we systematically identified BZR members in *N. benthamiana*, analyzed their expression patterns in different tissues and their gene expression profiles in response to drought, cold, heat, and salt stress. Our work lays the foundation for the future functional analysis of the *BZR* gene in *N. benthamiana*, and provides valuable information for a further understanding of the structure and expression of the *BZR* gene in solanaceous crops.

## 2. Results

### 2.1. Identification of BZR Gene Family Members in N. benthamiana

The BZR transcription factor contains a BES1_N superfamily domain, and we identified possible BZR members in *N. benthamiana* based on this standard. The amino acid sequences of the six identified Arabidopsis BZR members were downloaded from The Arabidopsis Information Resource (https://www.arabidopsis.org/ accessed on 22 September 2021), and the *N. benthamiana* genome sequence was downloaded from Sol Genomics Network (https://solgenomics.net/ accessed on 22 September 2021). After two rounds of BLASTP, a total of 14 *NbBZRs* were identified (Supplementary Table S1). These members were named from *NbBZR1* to *NbBZR14* according to their chromosomal positions. They ranged in length from 182 to 330 amino acids (aa) and most were about 300 aa long. Their calculated molecular weights were 20–35 kDa, and their isoelectric points were in the range from 6.89–9.39 (Table 1).

### 2.2. Phylogenetic Analysis of NbBZR Genes

**Figure 1:**
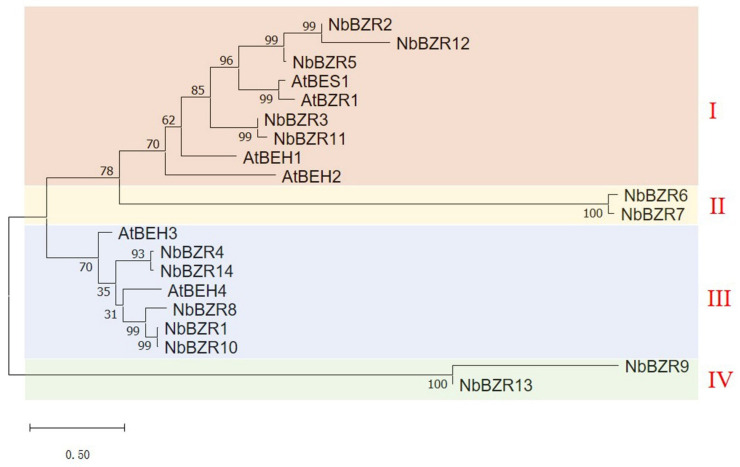
Phylogenetic tree of BZR transcription factors in *N. benthamiana* and *A. thaliana.* The phylogenetic tree was constructed using *BZR* amino acid sequences by the neighbor-joining method in MEGA X with 1000 bootstrap replicates. The phylogenetic tree was divided into four groups, which were shown in different colors, and identified by red Roman numerals.

In order to understand the relationships between the *BZR* family genes in Arabidopsis and *N. benthamiana*, a phylogenetic tree was constructed using the amino acid sequences of the six Arabidopsis and fourteen *N. benthamiana* members. The fourteen *NbBZR* genes could be divided into four groups, with five members in group I (*NbBZR2, -12, -5, -3, -11*), which also contained *AtBES1, AtBZR1, AtBEH1,* and *AtBEH2* (Figure 1). *NbBZR6* and *NbBZR7* form the second group (Figure 1). There are five members in group III (*NbBZR4, -14, -8, -1, -10*), and *AtBEH3, AtBEH4* are located in this group (Figure 1). Two members were contained in group IV, including *NbBZR9* and *NbBZR13,* which do not contain an Arabidopsis homolog (Figure 1).

### 2.3. Analysis of NbBZR Conserved Motifs, Gene Structure and Functional Domains

**Figure 2:**
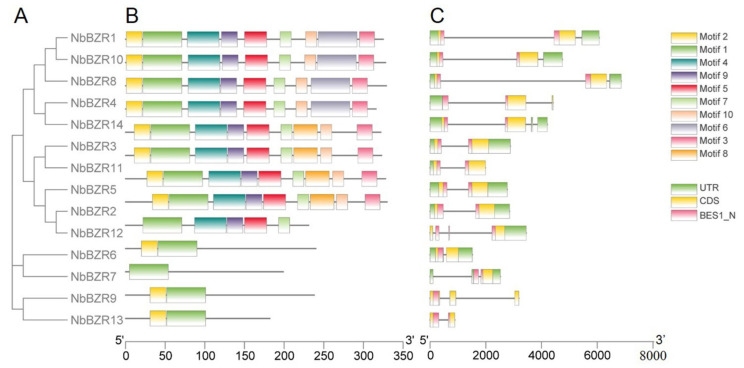
Analysis of conserved motifs, gene structure and domains in the *BZR* genes of *N. benthamiana.* (**A**) Phylogenetic tree constructed using the NbBZR protein sequences. (**B**) Ten types of conserved motifs are predicted in the NbBZR protein sequences. The different motifs are shown in different color boxes. The sequence information for each motif is provided in Supplementary Table S2. (**C**) The gene structure of *NbBZR* members (untranslated regions, exons, and introns are shown as light green boxes, yellow boxes and horizontal lines, respectively. The red boxes represent the BES1_N domain).

An online MEME analysis was next used to identify conserved motifs among the 14 *NbBZR* members, and a total of 10 conserved motifs were predicted (Supplementary Table S2). Most members contained 5–9 motifs, but *NbBZR6/-7/-9/-13* contained only two (Figure 2A,B). All 14 members contained the conserved BES1_N functional domain and most of them had two exons (Figure 2C).

### 2.4. Prediction of Cis-Elements in the NbBZR Promoters

**Figure 3:**
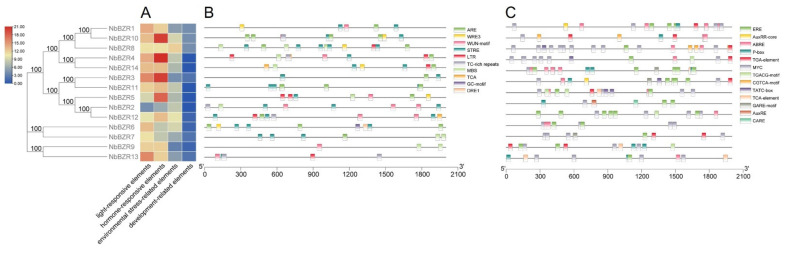
Prediction of cis-acting elements in the *NbBZR* promoter regions. (**A**) Schematic representation of the numbers of four types of cis-acting elements predicted in the promoter region of each *NbBZR* member. (**B**,**C**) The type, quantity and position of environmental stress-related elements (**B**) and hormone-response elements (**C**) in the *NbBZR* promoter region.

In order to further understand the possible regulatory mechanism of the *NbBZR* gene, we used the PlantCARE web server to search for possible cis-elements in the *NbBZR* 2000 bp promoter region. A total of 2164 cis-acting elements of 84 types were identified in the *NbBZR* promoter region (Supplementary Table S3). These cis-acting elements were related to environmental stress, hormonal response, development and light response, etc. (Figure 3A). A total of 101 elements related to environmental stress in the whole family were predicted in 10 categories, of which STRE, TCA and ARE elements were the most abundant (Figure 3B). A total of 187 hormone-related components were predicted in 13 categories, mainly related to JA, ethylene, and ABA (Figure 3C). This indicated that the *NbBZR* family genes may have been involved in a variety of stress and plant hormone response processes, and could effectively promote plant growth and stress resistance.

### 2.5. Expression Patterns of NbBZR Genes in Different Tissues

**Figure 4:**
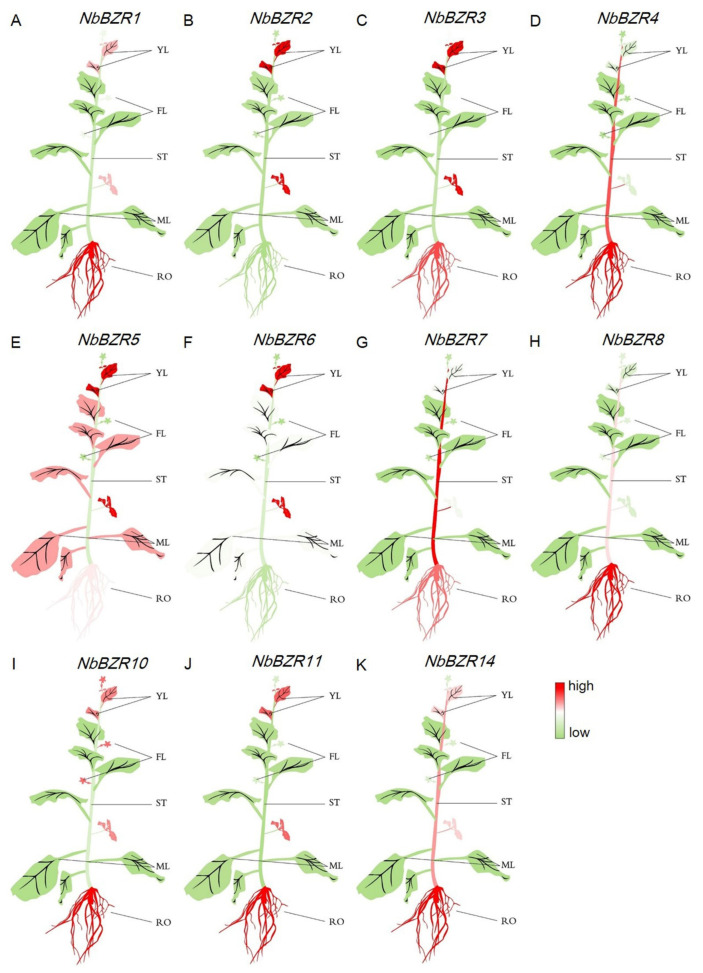
Expression levels of *NbBZRs* in different tissues (**A**–**K**). The mean expression values were calculated from three independent biological replicates relative to that in young leaves. YL: young leaf; MF: mature leaf; ST: stem; RO: root; FL: flower. Red represents a high expression level and green represents a low expression level. The raw data of relative expression values and standard errors are provided in Supplementary Figure S1.

We then analyzed the expression level of most *NbBZRs* in five different tissues (roots, stems, young leaves, mature leaves and flowers). The results from *NbBZR9/-12/-13* were not presented because their expression levels proved to be very low. *NbBZR5* was the only member that was highly expressed in mature leaves (Figure 4A,K and Supplementary Figure S1). *NbBZR4/-7/-8/-14* were expressed at high levels in the stem, especially *NbBZR7* (Figure 4A,K and Supplementary Figure S1). *NbBZR1/-3/-4/-5/-7/-8/10/-11/-14* were expressed at high levels in the roots but, except for *NbBZR10*, the *NbBZR* transcripts did not accumulate to high levels in flowers (Figure 4A,K and Supplementary Figure S1). These results indicated that the different *NbBZR*s were expressed differently in different tissues.

### 2.6. Expression Profiles of NbBZR Genes in Response to Abiotic Stress Treatments

**Figure 5:**
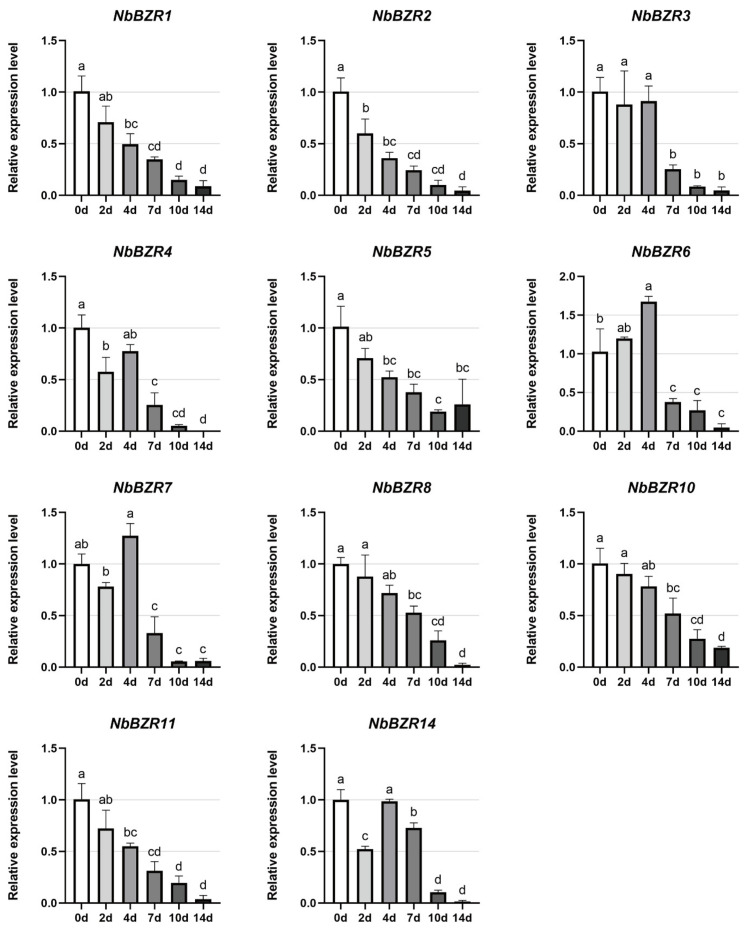
Expression analysis of *NbBZRs* after drought (0–14 days) treatment. Gene expression was normalized to control unstressed expression level, which was assigned a value of 1. Data represent the averages of three independent experiments ± SD. Standard errors are shown as bars above columns. Different letters indicate significant differences according to Tukey’s multiple comparisons test. Columns with different marked letters are significantly different (*p* < 0.05), and those with the same marked letter or share a letter are not significantly different.

We finally examined the expression patterns of the *NbBZR* gene family members to various types of abiotic stress: drought, cold, high temperature, and salt stress. For drought stress, samples were collected on days 0, 2, 4, 7, 10 and 14. All 11 tested genes were significantly down-regulated after 7 days of stress treatment (Figure 5) and levels generally remained low. There was some evidence that the expression of *NbBZR6* was slightly up-regulated in the early stages (2–4 days) of drought treatment (Figure 5).

**Figure 6:**
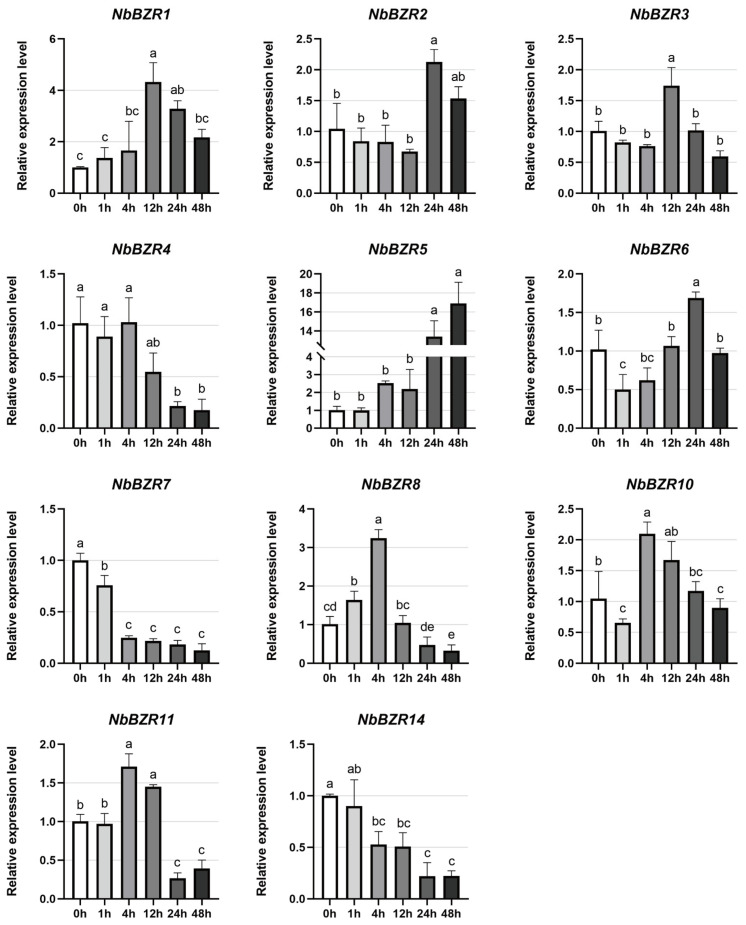
Expression analysis of *NbBZRs* after cold treatment (0–48 h). Gene expression was normalized to control unstressed expression level, which was assigned a value of 1. Data represent averages of three independent experiments ± SD. Standard errors are shown as bars above columns. Different letters indicate significant differences according to Tukey’s multiple comparisons test. Columns with different marked letters are significantly different (*p* < 0.05), and those with the same marked letter or share a letter are not significantly different.

There was a complex of *NbBZRs* that were subject to cold treatment. *NbBZRs 2, 3* and *5* were significantly up-regulated, most dramatically in *NbBZR5*, which showed a progressive up-regulation with time and was up-regulated 16-fold in 48 h relative to 0 h (Figure 6). *NbBZR4/-7/-14* showed a gradual downward trend as the stress time was prolonged. *NbBZR1/-8/-10/-11* were first up-regulated and then down-regulated, while *NbBZR6* was first down-regulated and then up-regulated (Figure 6).

**Figure 7:**
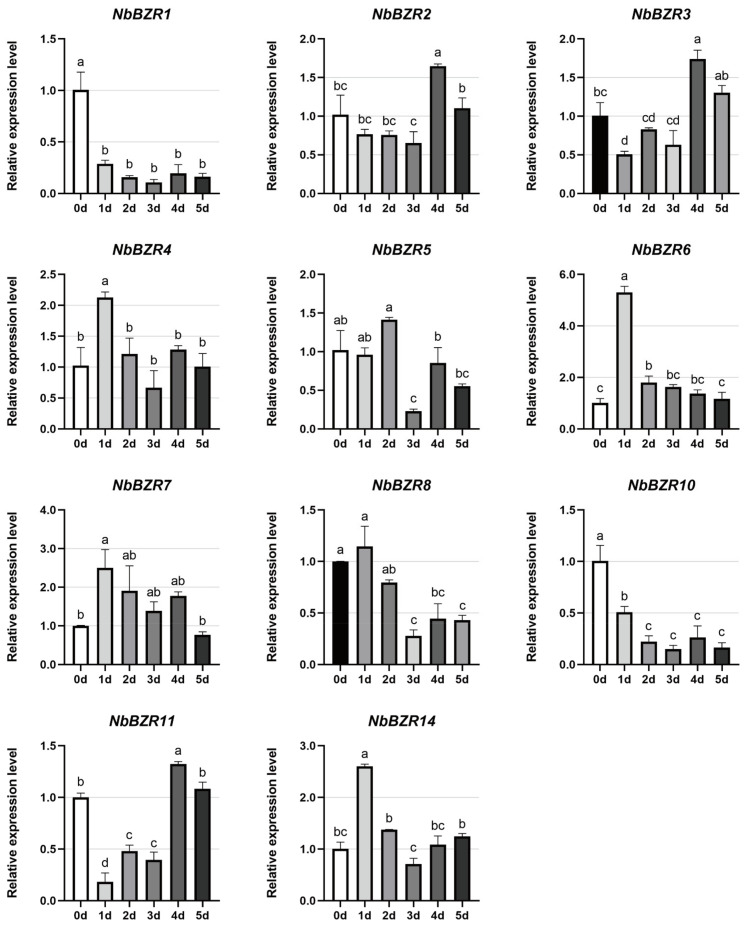
Expression analysis of *NbBZRs* after heat (0–5 days) stress. Gene expression was normalized to control unstressed expression level, which was assigned a value of 1. Data represent averages of three independent experiments ± SD. Standard errors are shown as bars above columns. Different letters indicate significant differences according to Tukey’s multiple comparisons test. Columns with different marked letters are significantly different (*p* < 0.05), and those with the same marked letter or share a letter are not significantly different.

During heat treatment, *NbBZR1/-8/-10* were strongly and rapidly inhibited (Figure 7) while *NbBZR2/-4/-6/-7* were induced to varying degrees, of which *NbBZR6* was the most induced, and from an early stage (Figure 7). The levels of *NbBZR3/-11* decreased on the first, second, and third days of heat treatment, and then began to increase (Figure 7).

**Figure 8:**
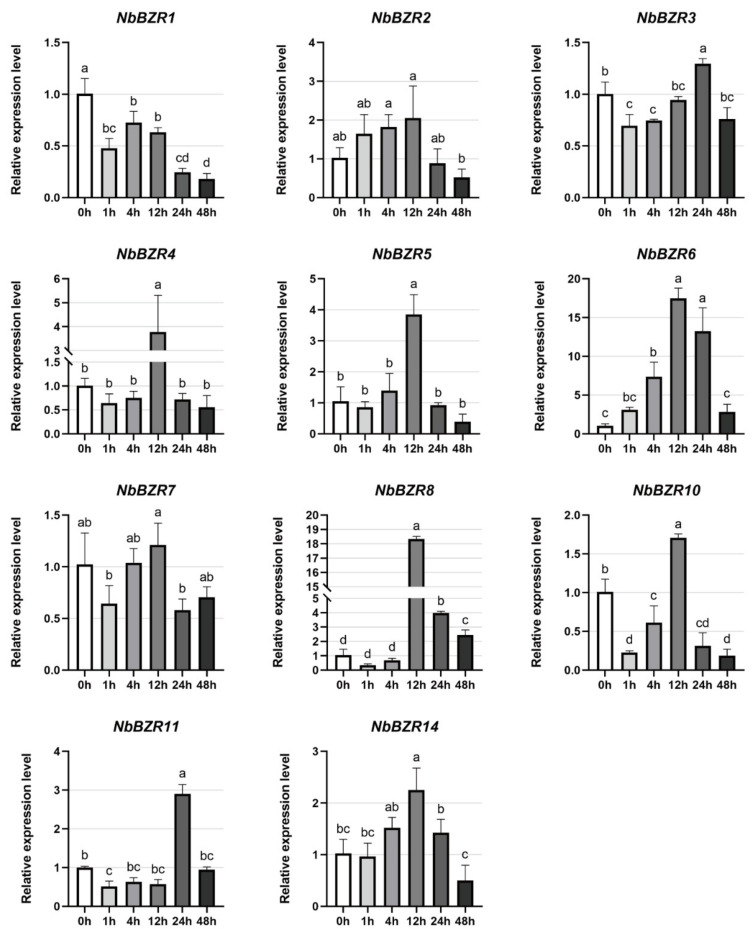
Expression analysis of *NbBZRs* after salt (0–48 h) treatment. Gene expression was normalized to control the unstressed expression level, which was assigned a value of 1. Data represent averages of three independent experiments ± SD. Standard errors are shown as bars above columns. Different letters indicate significant differences according to Tukey’s multiple comparisons test. Columns with different marked letters are significantly different (*p* < 0.05), and those with the same marked letter or share a letter are not significantly different.

Under salt stress, *NbBZR-4/-5/-6/-8/-11* were significantly induced, especially *NbBZR6* and -*8*, the expression of which increased about 18-fold after 12 h (Figure 8). *NbBZR1* was significantly down-regulated, decreasing to 20% of the control at 48 h (Figure 8). The expression levels of *NbBZR14* were slightly induced after the stress treatment, while *NbBZR2/-3* and *-7* were slightly inhibited (Figure 8).

## 3. Discussion

The BZR family is a small family of plant-specific transcription factors with important functions in plant growth and development and in BZR-mediated abiotic stress [30]. The BZR gene family members were systematically identified in Arabidopsis [13,14], *Brassica rap*a [31], sugar beet (*Beta vulgaris* L.) [33], maize (*Zea mays* L.) [32], apple (*Malus domestica)* [34], wheat (*Triticum aestivum* L.) [35] and seven legumes [30] but, prior to our study, there was no in-depth research report on the BZR TF family in *N. benthamiana*. In this work, a total of 14 *BZR* genes in the *N. benthamiana* genome were identified (Table 1). The 11 genes studied in detail were widely expressed at different levels in different tissues (Figure 4), suggesting that they may act as growth regulators. An analysis of the promoter region of the *NbBZR* gene revealed the existence of a variety of cis-acting elements to regulate the temporal and spatial expression levels of the genes (Figure 3). In addition to hormonal response elements, some elements related to stress and development were also identified (Figure 3). Following drought, cold, heat and salt stress, there were differential expression patterns among the *NbBZR* transcription factors (Figure 5, Figure 6, Figure 7 and Figure 8). Thus, the *NbBZR* gene may be a complex participant in the regulation of plant development and abiotic stress resistance pathways.

The transcript accumulation of all 11 tested genes was significantly down-regulated after 7 days of drought stress (Figure 5), so NbBZR may respond to drought stress in a negative manner. Previous studies showed that drought stress induces the production of ABA, which promotes stress tolerance [36], and that the BR signal and ABA pathways antagonize each other to coordinate plant growth and drought stress [37,38]. Therefore, the drought-induced, down-regulation of *NbBZR* expression may be the result of the activation of the ABA pathway. In addition, a previous study confirmed that the BR signaling pathway inhibits the drought response by regulating the mutual inhibition mechanism between BES1 and RD26 [21]. RD26 is a negative regulator of the BR pathway, and RD26 can be induced by drought to promote the expression of drought-regulating genes, thereby enhancing the drought resistance of plants. BES1 can inhibit the expression of drought-related genes such as RD26, leading to the suppression of drought response [21]. In our results, we observed that drought stress which inhibited *NbBZR* gene expression may also be caused by the induction of *RD26*. Therefore, we speculate that the knockdown of the *BZR* gene family of *N. benthamiana* may improve the drought stress resistance of plants. However, not all *BZR* genes in all species can be down-regulated under drought stress. For example, only one of the three *BZR* members tested after drought stress is down-regulated, while the other two are up-regulated in soybean [30]. In Medicago, among the six genes tested after drought stress, only two genes were down-regulated (*MtBZR2/5*), and the other genes had no effect [30]. In *Brassica rapa*, the expression of 10 *BrBZR* genes was up-regulated after drought stress, while only two genes continued to be down-regulated during the stress treatment [31]. It shows that the *BZR* genes of different species respond differently to the same kind of stress.

The response of *NbBZR* to other stresses (cold stress, heat stress, salt stress) is irregular, some are induced, and some are inhibited, which is consistent with the phenomenon observed in other species [30,31,32,34,35]. In our results, members with specific responses were observed in each stress. For example, *NbBZR5* increased 16-fold in 48 h under cold stress (Figure 6), and *NbBZR1/-10* was strongly inhibited under heat stress (Figure 7). During salt stress, *NbBZR6/-8* increased 18-fold in 12 h (Figure 8). These gene members may have important functions in the corresponding stress process and should be given priority in further functional identification.

The analysis of the expression results suggests that some *NbBZR* members participate in the response to multiple stresses. For example, the expression of *NbBZR1* was significantly inhibited under both heat and salt stress, whereas *NbBZR4/-6* were significantly induced under these treatments (Figure 7 and Figure 8). The expression of *NbBZR5* was up-regulated under both cold stress and salt stress (Figure 6 and Figure 8). It also appears that some members may respond in opposite ways to different abiotic stresses. For example, heat stress can inhibit the expression of *NbBZR8* (Figure 7), while salt stress can increase its expression (Figure 8). In a similar manner, cold stress can down-regulate the expression levels of *NbBZR4/-7/-14*, while heat stress can induce them (Figure 6 and Figure 7).

In this study, ten conservative motifs of *NbBZRs* are shown, and the motif composition of members in the same group are more similar (Figure 2). Motif-1 is an atypical bHLH DNA binding domain, which is the most conserved region in the BZR proteins, and the common motif in all BZR proteins [12]. Motif-4 has a serine-rich sequence (SxxxSxxxSxxx-SxxxS), which is considered to be a putative phosphorylation site for members of the GSK3 kinase family [39] and is also found in the *BZR* genes of Legume [30], *Brassica rapa* [31], and wheat [35]. Motif-8 is a PEST domain involved in the control of protein stability [12] and was also identified in the *BZR* genes of Legume [30] and *Brassica rapa* [31]. Motif-2 was identified in the *BZR* genes of apple [34] and *Zea mays* L. [32], and Motif-3/-5/-6/-10 were also found in the *BZR* genes of *Brassica rapa* [31].

In summary, these data suggest that the BZR transcription factor family in *N. benthamiana* plays a role in regulating plant physiology and morphology, and also has stress signal activity. The genome-wide identification and characterization of the members of the BZR TF family in *N. benthamiana* is an important starting point for the further in-depth study of the function of this gene family and can lay a foundation for the breeding and genetic improvement of solanaceous crops.

## 4. Materials and Methods

### 4.1. NbBZR Gene Identification

The protein sequences of the six Arabidopsis BZR genes were downloaded from The Arabidopsis Information Resource (https://www.arabidopsis.org/ accessed on 22 September 2021), and the genome of *N.* *benthamiana* was downloaded from the Sol Genomics Network (https://solgenomics.net/ accessed on 22 September 2021) [40]. The method used to identify all the putative *NbBZR* referred to Wu et al. [41]. First, all Arabidopsis BZR sequences were used to search for possible NbBZR sequences using TBtools [42]. Then, NCBI′s Batch CD-Search function was used to confirm whether the candidate NbBZR had a characteristic BES1_N superfamily domain (cl05316). Those candidates that did not meet the above conditions were eliminated. The predicted CDS length, isoelectric point, and molecular weight of the *NbBZR* genes were determined by ExPASy [43].

### 4.2. Phylogenetic Analysis

Phylogenetic analysis was performed based on the full-length protein sequences of AtBZR and NbBZR using the MEGA X program by the neighbor-joining (NJ) method, and a bootstrap test was carried out with 1000 iterations [44].

### 4.3. Analysis of Conserved Motifs, Gene Structure and Functional Domains

The conserved motifs of the genes were analyzed by the MEME program with a maximum number of motifs set to 10 [45]. Gene structures were analyzed and visualized by TBtools using *N. benthamiana* genome annotation file. Functional domains were analyzed and visualized using NCBI Batch CD-Search (https://www.ncbi.nlm.nih.gov/Structure/bwrpsb/bwrpsb.cgi accessed on 22 September 2021) and TBtools (V1.089).

### 4.4. Prediction of Cis-Elements in the Putative Promoter Regions

To investigate cis-elements in the promoter regions of all the obtained genes, we downloaded 2 kb of the genomic DNA sequences, upstream of the initiation codon (ATG) of each gene, from the Solanaceae genome database. The putative cis-regulatory elements in the promoter sequences were analyzed via PlantCARE (http://bioinformatics.psb.ugent.be/webtools/plantcare/html/ accessed on 22 September 2021) [46] and visualized by TBtools (V1.089).

### 4.5. Plant Materials and Abiotic Stress Treatments

In order to perform quantitative PCR expression analysis, *N. benthamiana* seeds were planted in greenhouse soil under the following controlled conditions: temperature 23 ± 1 °C, 14 h light/10 h night, and relative humidity 60%. *N. benthamiana* was transferred to stress treatment 21 days after planting. Drought stress consisted of stopping the normal watering regime for 14 d and leaves were sampled 0, 2, 4, 7, 10 and 14 days after the treatment started [30]. For salt stress, the plants were treated with 200 mM NaCl solution and the leaves were collected at 0, 1, 4, 12, 24 and 48 h after salt treatment [30]. For cold or heat treatment, the plants were subjected to 4 ± 1 °C [31] or 30 ± 1 °C [47] conditions, respectively. The leaves were collected at 0, 1, 4, 12, 24 and 48 h after cold treatment [31] and at 0, 1, 2, 3, 4 and 5 d after heat treatment [47,48]. All collected samples from three biological replicates were quickly frozen in liquid nitrogen and stored at − 80 °C. RNA was extracted to analyze the expression patterns of the different BZR transcription factors.

### 4.6. RNA Isolation and Expression Analysis of NbBZR Genes

Total RNA was extracted by the TRIZOL method, and the first cDNA strand was generated using Toyobo cDNA First Strand Synthesis Kit (Toyobo, Shanghai, China). Then, the RT-qPCR was performed on the Roche LightCycler^®^480 Real-Time PCR instrument (Rotkreuz, Switzerland) with Toyobo Premix Kit (Toyobo, Shanghai, China). Three independent biological replicates and three technical replicates were adopted. The *N. benthamiana ubiquitin-conjugating enzyme E2* (*UBC*) gene was used as the internal reference gene to calculate relative gene expression levels using the 2^−ΔΔ C(t)^ method [49]. All primers are listed in the Supplementary Table S4.

## 5. Conclusions

This study systematically identified the BZR TF members in *N. benthamiana*, and analyzed their tissue-specific expression and expression profiles in response to four abiotic stresses. The results indicated that *NbBZR* may have had a variety of resistance-related functions. Therefore, our findings in this study will help to select suitable candidate genes for further functional identification.

## Figures and Tables

**Table 1:** Detailed information of the 14 predicted BZR proteins in *N. benthamiana.*

Gene Symbol	Gene Locus	Gene Position	Chromosome Location	Strand	CDS (bp)	Protein Length (aa)	Molecular Weight(kDa)	Theoretical PI
*NbBZR1*	Niben101Scf00073g05010.1	Niben101Scf00073	Niben101Scf00073:539113,545181	+	981	327	35.27	7.65
*NbBZR2*	Niben101Scf00219g06001.1	Niben101Scf00219	Niben101Scf00219:603740,606595	+	990	330	35.27	8.44
*NbBZR3*	Niben101Scf00894g00004.1	Niben101Scf00894	Niben101Scf00894:692,3575	−	966	322	34.59	9.05
*NbBZR4*	Niben101Scf01983g15010.1	Niben101Scf01983	Niben101Scf01983:1485193,1491375	−	987	329	35.43	9.39
*NbBZR5*	Niben101Scf03110g05009.1	Niben101Scf03110	Niben101Scf03110:623795,626566	+	984	328	35.08	9.34
*NbBZR6*	Niben101Scf03282g02006.1	Niben101Scf03282	Niben101Scf03282:277240,278755	+	720	240	24.74	9.37
*NbBZR7*	Niben101Scf03729g00010.1	Niben101Scf03729	Niben101Scf03729:20537,23061	−	597	199	20.59	9.33
*NbBZR8*	Niben101Scf04132g01002.1	Niben101Scf04132	Niben101Scf04132:171903,178765	+	984	328	35.70	7.58
*NbBZR9*	Niben101Scf05540g00014.1	Niben101Scf05540	Niben101Scf05540:26876,39192	+	714	238	26.85	9.27
*NbBZR10*	Niben101Scf05948g02026.1	Niben101Scf05948	Niben101Scf05948:259103,263853	−	975	325	35.10	8.44
*NbBZR11*	Niben101Scf06112g01006.1	Niben101Scf06112	Niben101Scf06112:109855,111842	+	969	323	34.85	9.16
*NbBZR12*	Niben101Scf10412g00006.1	Niben101Scf10412	Niben101Scf10412:11951,15403	−	693	231	24.34	6.89
*NbBZR13*	Niben101Scf10887g01009.1	Niben101Scf10887	Niben101Scf10887:126261,127150	+	546	182	20.79	8.47
*NbBZR14*	Niben101Scf12841g03023.1	Niben101Scf12841	Niben101Scf12841:315425,319633	+	948	316	33.93	9.18

Notes: The signs “+” and “−“ signify sense and anti-sense transcription respectively.

## Supplementary Materials

The following are available online at https://www.mdpi.com/article/10.3390/ijms221910379/s1.
