# Supplementary figures and images for: Genome-Wide Identification and Expression Profiling of the BZR Transcription Factor Gene Family in Nicotiana benthamiana
Source: Int J Mol Sci. 2021 Sep 26;22(19):10379. doi: 10.3390/ijms221910379 (PMC8508657; doi:10.3390/ijms221910379)

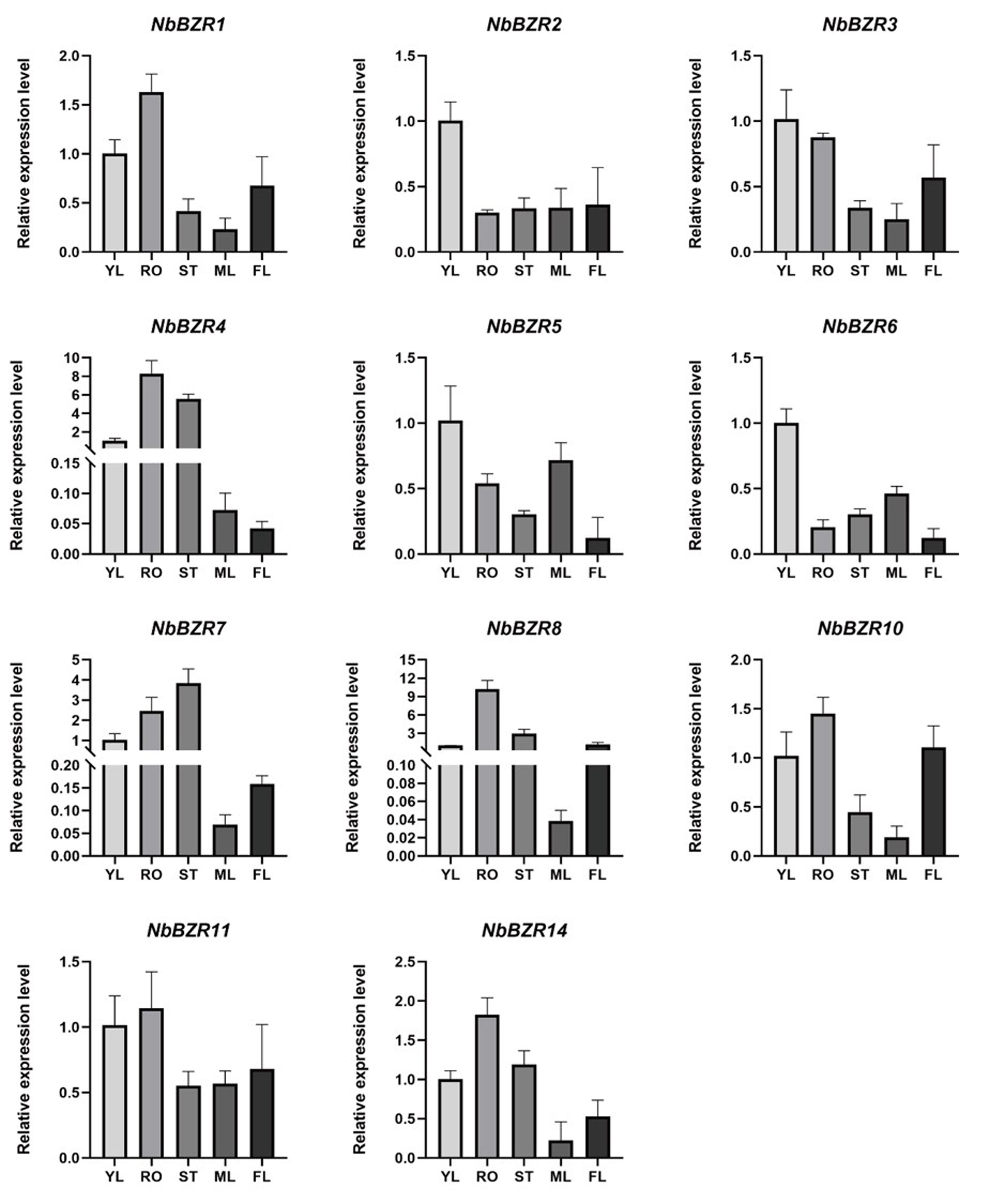

Supplement: Supplementary file 1 [file ijms-22-10379-s001.zip › Supplementary Figure S1.jpg]
